# Tissue-Specific Dynamics in the Endophytic Bacterial Communities in Arctic Pioneer Plant *Oxyria digyna*

**DOI:** 10.3389/fpls.2020.00561

**Published:** 2020-05-13

**Authors:** Cindy Given, Elina Häikiö, Manoj Kumar, Riitta Nissinen

**Affiliations:** ^1^Department of Biological and Environmental Science, University of Jyväskylä, Jyväskylä, Finland; ^2^Department of Environmental and Biological Sciences, University of Eastern Finland, Kuopio, Finland

**Keywords:** endophytic bacteria, *Oxyria digyna*, tissue-specificity, bacterial succession, pioneer plant, arctic bacteria

## Abstract

The rapid developments in the next-generation sequencing methods in the recent years have provided a wealth of information on the community structures and functions of endophytic bacteria. However, the assembly processes of these communities in different plant tissues are still currently poorly understood, especially in wild plants in natural settings. The aim of this study was to compare the composition of endophytic bacterial communities in leaves and roots of arcto-alpine pioneer plant *Oxyria digyna*, and investigate, how plant tissue (leaf or root) or plant origin affect the community assembly. To address this, we planted micropropagated *O. digyna* plants with low bacterial load (bait plants) in experimental site with native *O. digyna* population, in the Low Arctic. The endophytic bacterial community structures in the leaves and roots of the bait plants were analyzed after one growing season and one year in the field, and compared to those of the wild plants growing at the same site. 16S rRNA gene targeted sequencing revealed that endophytic communities in the roots were more diverse than in the leaves, and the diversity in the bait plants increased in the field, and was highest in the wild plants. Both tissue type and plant group had strong impact on the endophytic bacterial community structures. Firmicutes were highly abundant in the leaf communities of both plant types. Proteobacteria and Bacteroidetes were more abundant in the roots, albeit with different relative abundances in different plant groups. The community structures in the bait plants changed in the field over time, and increasingly resembled the wild plant endophytic communities. This was due to the changes in the relative abundances of several bacterial taxa, as well as species acquisition in the field, but with no species turnover. Several OTUs that were acquired by the bait plants in the field and represent phosphate solubilizing and diazotrophic bacterial taxa, suggesting major role in nutrient acquisition of these bacteria for this nonmycorrhizal plant, thriving in the nutrient poor arctic soils.

## Introduction

All eukaryotes maintain a close relationship with diverse microorganisms. Rapidly accumulating data indicates, that plant microbiome is a key determinant of plant health and productivity by providing a plethora of functional capacities (Brader et al., 2014; Hardoim et al., 2015). Numerous studies have shown that soil is the main source of endophytic bacteria and that root endophytes are often seen as a subset of rhizosphere bacteria (Bodenhausen et al., 2013). Attracted by root exudation, different bacteria migrate to the rhizosphere and rhizoplane before penetrating into plant roots, from where they may also colonize aboveground plant tissues (reviewed by Compant et al., 2010). Phyllosphere is another route for endophytic bacterial colonization, with air and rain considered to be sources of endophytic bacteria (Hardoim et al., 2015). Abiotic and biotic factors influence the assembly of rhizospheric, phyllospheric, and endophytic bacterial communities. Factors such as soil type (Conn and Franco, 2004; Bulgarelli et al., 2012), host plant species (Nissinen et al., 2012; Ding et al., 2013), plant age and genotype (Marques et al., 2014; Wagner et al., 2016), as well as plant developmental stage (Chaparro et al., 2014; Yuan et al., 2015) have all been shown to have a major effect on the endophytic bacterial community composition. Plant hormones such as salicylic acid or jasmonic acid also shape microbial communities in the rhizosphere and root endosphere (Haney and Ausubel, 2015; Lebeis et al., 2015). Further, different plant tissues provide specific biotic and abiotic conditions which might select for specific microbiota assemblages (Mengoni et al., 2014; Bai et al., 2015; Checcucci et al., 2016). The reported endophytic bacterial population densities are higher in roots (10^5^–10^7^ CFU g^–1^ of fresh weight) than in the leaves (10^3^–10^4^ CFU g^–1^), likely reflecting the higher nutrient levels and less stressful conditions in the roots (reviewed by Compant et al., 2010).

Endophytic bacteria have been studied extensively, but relatively little is still known about the succession and factors regulating the colonization process of the endophytic bacteria in different plant tissues. The available studies on the succession of phyllosphere microbiome (Copeland et al., 2015), rhizosphere microbiome (Chaparro et al., 2014), and root endophytic communities (Yuan et al., 2015) focus on agricultural plants in temperate climate. In contrast, only a few studies have focused on wild plants in high-stress environments like the Arctic, alpine treelines, or deserts (Nissinen et al., 2012; Coleman-Derr et al., 2016; Kumar et al., 2017a; Carper et al., 2018), and no data is currently available on plant colonization by microbes in these biomes.

Plants in the Arctic have to cope with several abiotic stressors including a short and cold growing season and strong annual fluctuations in light and temperatures (Wilson, 1954). Nutrient accumulation and recycling in the Arctic is very slow. The majority of nutrients are in plant inaccessible forms, and pioneer soils with low organic contents often have negligible levels of, e.g., nitrogen or phosphorus (Nadelhoffer et al., 1992). The target plant in this study, *Oxyria digyna* Hill (Mountain sorrel) is a perennial pioneer species common in glacier forefront communities and successful in colonizing mineral soils with very low nutrient levels. As a non-mycorrhizal plant species with hemiarctic and alpine distribution, *O. digyna* is a good model plant for dissecting the role of endophytic microbiota in plant fitness in demanding conditions like the Arctic. For mycorrhizal plants, the mycorrhizal partner (with its associated bacteria) has a major role in plant nutrition and stress tolerance, while for a non-mycorrhizal plant like *O. digyna* other endo- and rhizospheric microbes likely are of major importance. *O. digyna* is also amiable for manipulative studies, and more likely to yield biologically relevant findings in such settings compared to mycorrhizal species.

Our previous studies have demonstrated, that the structure of bacterial communities associated with *O. digyna* are different across different compartments (root endosphere, rhizosphere, and bulk soils) (Kumar et al., 2016, 2017a). Interestingly, several taxa, e.g., Oxalobacteraceae, Comamonadaceae have been consistently detected in the root endosphere samples from three different climatic regions (alpine, low-arctic, and high-arctic) (Kumar et al., 2017a) as well as isolated from *O. digyna* vegetative tissues (Nissinen et al., 2012), and seeds (Given et al., unpublished), forming the core microbiome of *O. digyna*. Several of these core taxa (Comamonadaceae, *Clostridia* sp.) have also been shown to be potential nitrogen fixers (Kumar et al., 2017b). However, no information is currently available on the leaf bacterial communities of this perennial plant, or on factors determining plant tissue colonization in *O. digyna*.

The aims of this study were to dissect the factors impacting the assembly of endophytic bacterial communities in *O. digyna* plants. More specifically, we wanted to (1) compare the impact of plant origin and plant tissue type in community dynamics and to find out if (2) these endophytic communities are acquired and assembled in a similar way in transplanted tissue propagated plants and native plants growing in the same site, and (3) if these bacteria are equally distributed throughout the plant organs (leaves and roots). To that end, we brought micropropagated plantlets with a low initial microbial load to the field and compared the bacterial communities in the roots and leaves of these plants after one growing season and after overwintering, to those of native *O. digyna* plants growing in the field site.

## Materials and Methods

### Micropropagated *O. digyna* Plantlets and Acclimatization

The micropropagated *O. digyna* plantlets used in this experiment were initiated from seeds obtained from Kilpisjärvi region and propagated at the University of Oulu Botanical Garden for 54 months before the experiment. For this experiment, the plants were maintained at the University of Eastern Finland, Kuopio (62°53′30.2″N and 27°38′04.8″E). The plantlets were maintained under sterile conditions in half-strength Murashige and Skoog (MS) agar medium (Murashige and Skoog, 1962) in low light (30 μmol m^–2^ s^–1^) with a photoperiod of 16:8 h light:dark cycle and 21°C constant temperature. Forty-five days before transplantation to the field, the plants were transferred from the half-strength MS agar to sterile containers with vermiculite to allow root growth, and were watered with sterile 1/4 strength Hoagland’s solution (Hoagland and Arnon, 1950) in micropropagation room under similar conditions as above. The plants were then moved to greenhouse for acclimation, and were maintained in ambient conditions (21°C/18°C day/night temperature with the light level of 250 μmol m^–2^ s^–1^ and 16:8 h light:dark photoperiod) for 7 days. The lid was gradually opened to allow cuticle formation of plant leaves. After acclimation, individual plants were transferred into 7-cm-diameter net pots lined with 15 μ-mesh size plankton net and filled with washed and double-sterilized sand. The pots were acclimated outside on desks, under mesh fabric for 10 days before shipping to experimental site in Kilpisjärvi.

### Experimental Site and Sampling

**FIGURE 1:**
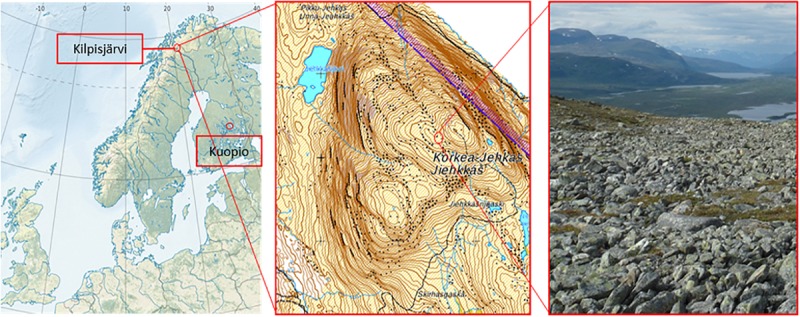
Locations of the plant propagation site in Kuopio (62°53′30.2″N and 27°38′04.8″E) and the study site in Jehkas fell area, Kilpisjärvi, North-Western Finland (69°1′N and 20°50′E).

The field site is located in low-arctic climate zone on fell Korkea-Jehkas at 925 m.a.s. (69°1′N and 20°50′E) in Kilpisjärvi area, North-Western Finland (Figure 1). The growing season in Kilpisjärvi is 80–100 days, and the annual mean temperature is −2.3°C. The soil in the field site is low organic matter moraine soil with patchy vegetation, dominated by *O. digyna*, *Saxifraga oppositifolia*, mosses and lichens. The site is located on a slope next to snowmelt stream which keeps the soil moist throughout the summer. In early July 2013, after the acclimation, six micropropagated plants were harvested (hereafter referred to as “starter plants”). The rest of the plants, a total of 65 pots of micropropagated plants (referred to hereafter as “bait plants”) were transported to the study site, planted in net pots next to a group of wild plants and covered with metal cages to protect the plants from reindeer grazing. The plants inside their net pots with 15 μ-mesh size plankton net lining were planted in soils, allowing free movement of soil water, bacteria and fungi to the bait plants. Twenty pots were harvested 50 days after planting, at the late growing season (late August) (referred to hereafter as the “August bait plants”). Simultaneously, 10 native *O. digyna* plants growing adjacent to the experimental plants were also harvested (referred to hereafter as the “wild plants”). The rest of the experimental plants were left in the study site for overwintering and harvested in mid-July 2014 (referred to hereafter as the “over-wintered bait plants” or OW). No wild plants were harvested at this time point. All plant samples were brought back to the laboratory and processed within 48 h after harvesting. A total of five replicates from starter plants, 10 replicates from August bait plants, 10 replicates from over-wintered bait plants, and 10 replicates from wild plants were used for analyses and statistical comparisons.

The soil temperatures at the experimental site reached 0°C at 5.6.2013 and varied between 4.5 and 11.0°C during the growing season. The soil temperatures dropped below 0°C on 24.9.2013, and varied between −0.5 and −2.5°C until reaching 0°C at 8.7.2014.

### Sample Treatment

The bait plants were carefully removed from the pots, and all plant samples were washed with running tap water to remove soil. Shoot and root samples were separated and surface-sterilized as described in Nissinen et al. (2012), with slight modifications: plant tissues were submerged into 2.5% sodium hypochlorite (NaOCl) for 3 min, then in 1% sodium thiosulfate (Na_2_H_2_O_3_) for 3 min, followed by three washes in sterile distilled water for 2 min each, and then tap dried on sterile tissue paper. Sterility checks were performed by plating the distilled water from the last washing step after every five samples on R2A, pH 6.5 medium. The plates were checked after 3–7 days and the samples that belong to the plates with no bacterial growth will be included in the study. Samples of surface-sterilized leaf and root samples (approximately 100 mg) were stored at −80°C until DNA isolation.

### DNA Extraction, PCR Amplification, and Amplicon Library Preparation

The metagenomic DNA was extracted from the plant samples with the Invisorb^®^ Spin Plant Mini Kit (Stratec Biomedical) according to the manufacturer’s instructions with slight modifications: the frozen samples were pre-homogenized with sterile mortar and pestle with liquid nitrogen to break the plant tissues. The frozen powder (homogenized plant samples) were then transferred into 2 ml sterile cryotube vials filled with a small portion (approximately 30 μl) of sterile 0.1 mm glass beads, 400 μl of lysis buffer, and 20 μl proteinase K (provided with the kit). The samples were always kept cool to prevent the damage of the eubacterial DNA from the plant enzymes released during the pre-homogenization. The samples were then homogenized again with bead-beater at maximum speed for 2 × 30 s with 30 s of a cooling period in between. This process is to enhance the breaking of bacterial cells. After homogenization, the DNA was extracted according to the manufacturer’s instructions. The DNA extracts were assessed for quality and quantity with NanoDrop^®^ ND-1000 spectrophotometer and DNA concentration was adjusted to 25–30 ng μl^–1^.

We used nested PCR approach and M-13 barcode PCR procedure (Mäki et al., 2016) for amplicon library preparation. First PCR round was performed with primer pair 799F (5′-AAC MGG ATT AGA TAC CCK G-3′) and 1492R (5′-GGY TAC CTT GTT ACG ACT T-3′) targeting the V5–V9 regions of 16S rRNA gene (Chelius and Triplett, 2001). PCR reaction for each sample contained 25–30 ng of DNA, 1× PCR buffer, 1 mg ml^–1^ of BSA, 0.2 mM of dNTP’s, 0.3 mM of each primer, and 1,250 U ml^–1^ Promega GoTaq^®^ DNA Polymerase in a total of 30 μl. The amplification was done in C1000^TM^ Thermocycler (Bio-Rad) with conditions: denaturation at 95°C for 3 min followed by 35 cycles of denaturing at 95°C for 45 s, annealing at 54°C for 45 s, and extension at 72°C for 1 min with the final extension at 72°C for 5 min. This step was done in duplicates. The PCR products were checked on 1% (w/v) agarose gel before pooling the products from the same samples together to be used as the template for the second step PCR.

M13-1062F (5′-TGT AAA ACG ACG GCC AGT – GTC AGC TCG TGY YGT GA-3′) and 1390R (5′-ACG GGC GGT GTG TRC AA-3′) were used for the second PCR step. Each 30 μl of PCR reaction contained 1 μl of the pooled amplicons from the previous step (1st PCR), 1× PCR buffer, 0.2 mM of dNTP’s, 0.3 mM of each primer, and 1,250 U ml^–1^ Promega GoTaq^®^ DNA Polymerase. The condition of PCR amplification was as described for the first PCR with 35 cycles. The PCR products were checked on 1% (w/v) agarose gel prior to continuing with the last step PCR.

Sample specific barcodes and P1-adaptor were attached in the third PCR round, using primers Barcode-M13 [5′-(BC1-48) – TGT AAA ACG ACG GCC AGT-3′] and 1390R-P1 (5′-CCT CTC TAT GGG CAG TCG GTG AT – ACG GGC GGT GTG TRC AA-3′). One PCR reaction (30 μl) contained 1 μl of the 1:1 diluted PCR product from the previous step (2nd PCR), 1× PCR buffer, 0.2 mM dNTP’s, 0.3 mM of each primer, and 1,250 U ml^–1^ Promega GoTaq^®^ DNA Polymerase. The PCR conditions were as in the first and second PCR but with only seven cycles of denaturation, annealing, and extension instead of 35 cycles.

The final PCR products were purified using Agencourt AMPure XP solution (Beckman Coulter) according to the manufacturer’s protocol. Agilent 2200 TapeStation (Agilent Technologies) was used to analyze the concentration of the eubacterial amplicons from the final PCR. The samples were then pooled equimolarly at the quantity of 20 ng of eubacterial DNA per sample, and then size fractionated to get the fractions of eubacterial amplicons at 350–620 bp using Pippin Prep (Sage Science). The collected fractions were purified with AMPure XP, and quantified using Agilent 2200 TapeStation. Two pools of 400 ng pool^–1^ were sequenced on Ion-torrent PGM with Ion PGM Hi-Q sequencing kit following manufacturer’s instructions at the University of Oulu (Finland) sequencing facility.

### Raw Sequence Processing and Data Analyses

The raw DNA sequencing data were processed using open-sourced bioinformatics pipelines on Quantitative Insights Into Microbial Ecology (QIIME) (Caporaso et al., 2010) and UPARSE (Edgar, 2013) based on the 16S rRNA gene data analysis pipeline developed by Pylro et al. (2014) with slight modifications in the quality filtering step. Low-quality reads (Q score <25) and short base pair length (<150 bp) sequences were removed. All the remaining reads were trimmed at 250 bp, clustered and aligned at 97% identity using USEARCH algorithm (Edgar, 2010). The individual operational taxonomic units (OTUs) were assigned taxonomies at 97% identity using UCLUST algorithm and RDP database (Wang et al., 2007). For alpha diversity analysis, all samples were rarefied to 500 reads per sample. Univariate Diversity Indices (DIVERSE, PRIMER 6, Quest Research Limited) was used to obtain the species richness, evenness and Shannon diversity index. The differences between the plant tissues and plant groups were tested separately for each indices using two-way ANOVA (SPSS Statistics, IBM).

The data was standardized as described in deCárcer et al. (2011), by rarefying the samples with more than the median reads to the median (1,744 reads), while the samples with fewer reads were used as such. This normalized data was used for the community structure and other analyses. Additionally, all OTUs with less than 30 reads, along with OTUs affiliated to chloroplast and mitochondria were removed before subsequent analyses. The influence of the tissue, plant group and plant type on bacterial community structures, based on Bray–Curtis distance matrices of square root transformed abundance data to quantify the community compositional differences, were analyzed using Permutational MANOVA (PERMANOVA) and visualized by principle component analysis (PCoA) ordinations at the OTU level. All these analyses were performed using PRIMER 6 software package with PERMANOVA+ add-on (PRIMER-E, Quest Research Limited) (Anderson, 2017). Venn diagram (Oliveros, 2007) was also used to visualize the group-specific and shared OTUs between samples.

For the differential abundance testing of the community members at the order level between plant tissues and between plant groups, we performed Kruskal–Wallis test with the log-transformed [log (X+1)] relative abundance data using RStudio statistical software (version 1.0.136). For Kruskal–Wallis test, only OTUs present in at minimum three samples were included.

Two treatments were regarded in the analysis, plant tissues and plant groups. Tissue consisted of leaf and root while plant groups including starter plants, August bait plants, over-wintered bait plants and wild plants.

## Results

### Endophytic Bacterial Diversity Is Higher in the Roots Than in the Leaves and Increases With Time in the Field

A total of 257,979 high quality sequence reads were obtained, and were clustered into OTUs at 97% sequence identity. Plastid and mitochondrial OTUs and OTUs with less than 30 reads were removed resulting in total 78,888 reads (1,111 ± 56 sequences per sample). These reads were assigned into a total of 196 OTUs, and used in further analysis.

**FIGURE 2:**
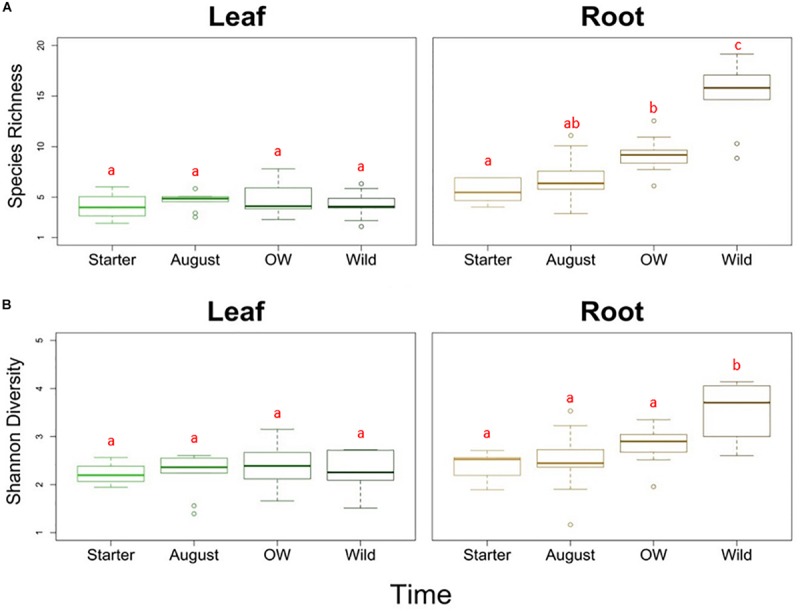
**(A)** Species richness and **(B)** Shannon diversity of endophytic bacterial communities in different tissues (leaf and root) and plant groups bait plants before field (Starter), bait plants after field (August), over-wintered bait plants (OW), and wild plants (Wild). Two-way ANOVA (*p* < 0.05) was used to determine the difference in the species richness and Shannon diversity between tissues and plant groups. Letters indicate the results from Tukey’s “Honest Significant Difference” test, where groups indicated with the same letter do not differ significantly.

Analyses of species richness and Shannon diversity of leaf communities revealed no difference between plant groups (two-way ANOVA, species richness: *p* = 0.676, Shannon diversity: *p* = 0.456; Figures 2A,B). On the other hand, the species richness was significantly different between plant groups in the roots (two-way ANOVA, *p* < 0.01; Figure 2A). The species richness in the starter roots was significantly lower than in the over-wintered and wild roots, while the richness in August roots did not differ significantly from starter and over-wintered roots (Tukey HSD). With regard to diversity, the starter, August and over-wintered roots had all significantly lower diversity levels than wild root samples (two-way ANOVA, *p* < 0.01, *c-hoc*: Tukey HSD; Figure 2B).

### Plant Tissue Type Is the Primary Determinant of the Endophytic Bacterial Community Structure

**FIGURE 3:**
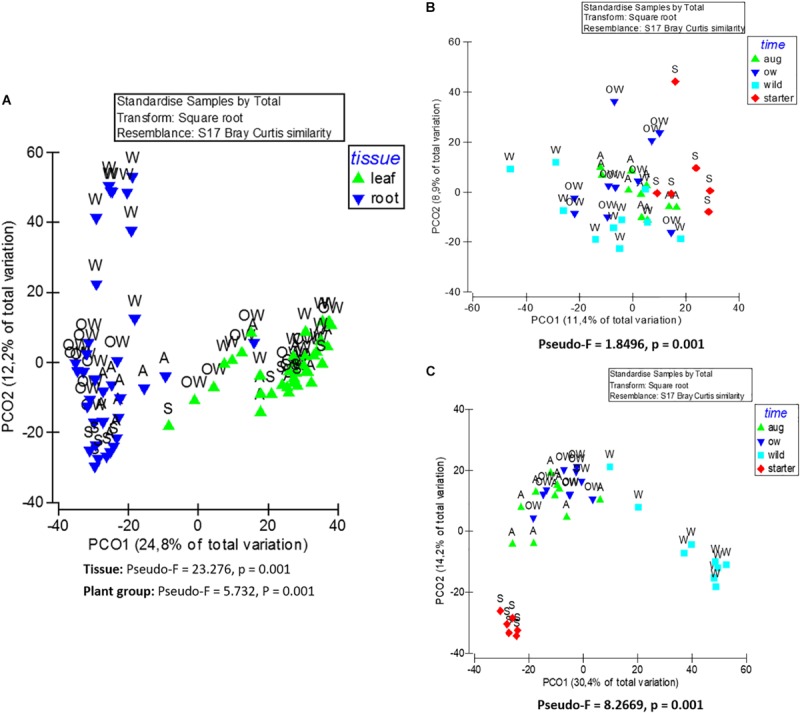
Principle coordinate analysis (PCoA) of endophytic bacterial (16S rRNA) community composition from **(A)** leaf and root samples, **(B)** root samples, and **(C)** leaf samples of *O. digyna.* The analyses are based on Bray–Curtis similarity matrix, and were performed with PRIMER software version 6. The pseudo-*F* and *p* values were obtained from PERMANOVA. S, starter = bait plants before field; A, aug = bait plants after field; OW = over-wintered bait plants; and W, wild = wild plants.

The endophytic bacterial communities were primarily shaped by tissue type (leaf or root) (PERMANOVA; Tissue: Pseudo-*F* = 23.276, *p* = 0.001). This was also visible in principle component analysis (PCoA) of community structures, where the communities clustered primarily according to the tissue type (Figure 3A).

**FIGURE 4:**
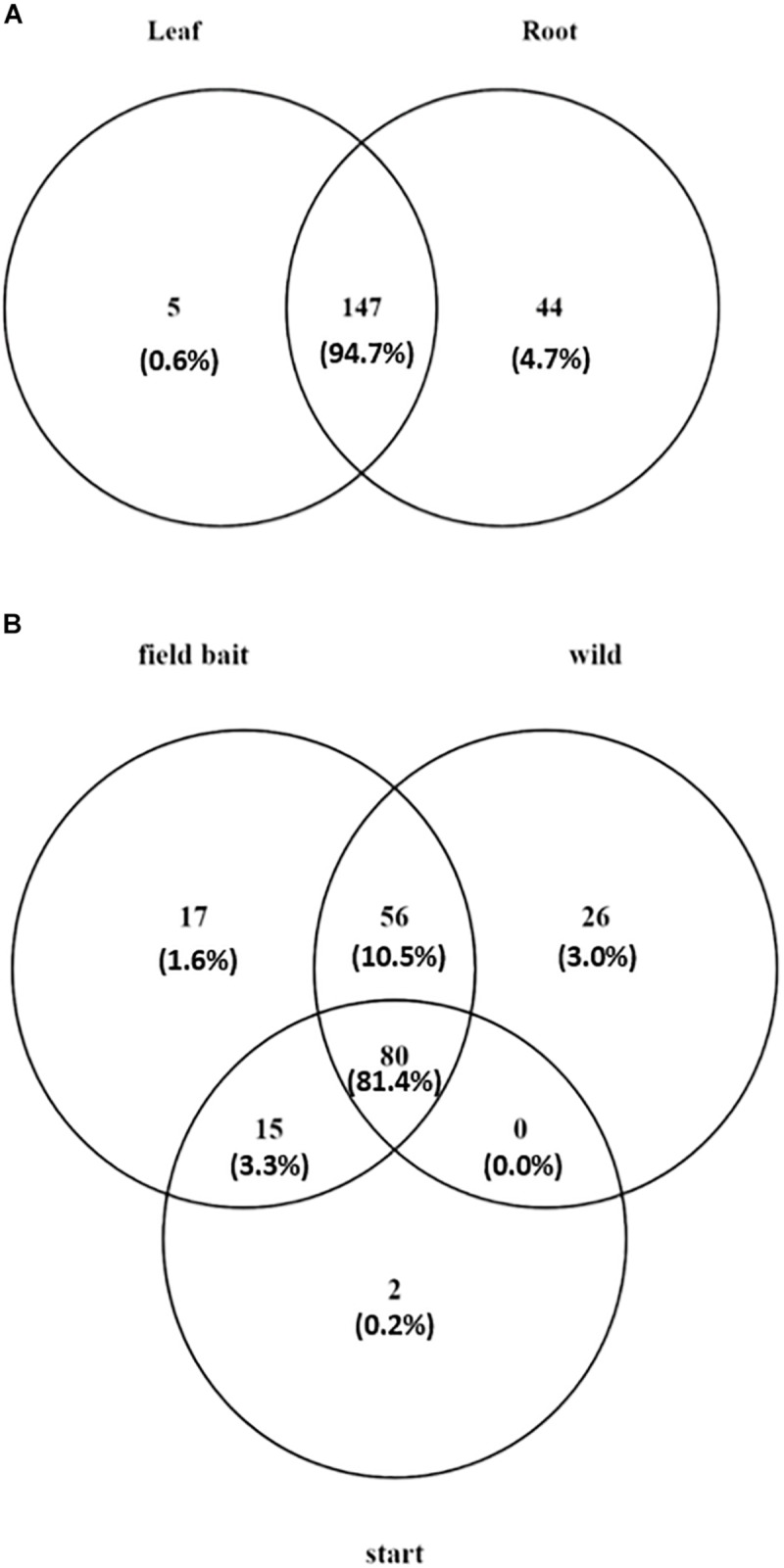
Venn diagram showing **(A)** number of tissue specific OTUs and OTUs present in both tissues in *O. digyna*, with %relative abundance of total sample set reads in parentheses, **(B)** number of OTUs present in different sample groups with %relative abundance of total sample set reads in parentheses. Field bait group combined the August bait plants and over-wintered bait plants. OTUs were clustered at 97% similarity level.

Overall, the majority of the OTUs were shared between leaf and root communities (147 OTUs and 94.7% of the total reads in the dataset; Figure 4A). However, 44 OTUs (4.68% of the total reads) were detected only in roots and five OTUs (0.59% of the total reads) were present only in leaves (Figure 4A) (list of tissue-specific OTUs in Supplementary Table S1).

Similarity percentages (SIMPER) by species contribution analysis identified the major OTUs contributing to the dissimilarity between the leaf and root communities (Supplementary Table S1). The most abundant OTU in the dataset, OTU1 (*Aeribacillus* sp.), was highly enriched in the leaf communities in all plant groups, with over 80% of OTU reads in the leaf samples. Also, several Alphaproteobacterial OTUs representing Sphingomonadales, Pseudomonadales, and Enterobacteriales were enriched in the leaf communities. In contrast, several OTUs representing Burkholderiales, including OTU2 (Oxalobacteraceae) and OTU6, OTU7, OTU12 (Comamonadaceae) and two OTUs (OTU3 and OTU25) representing *Flavobacterium* sp. were more abundant in the root communities.

**FIGURE 5:**
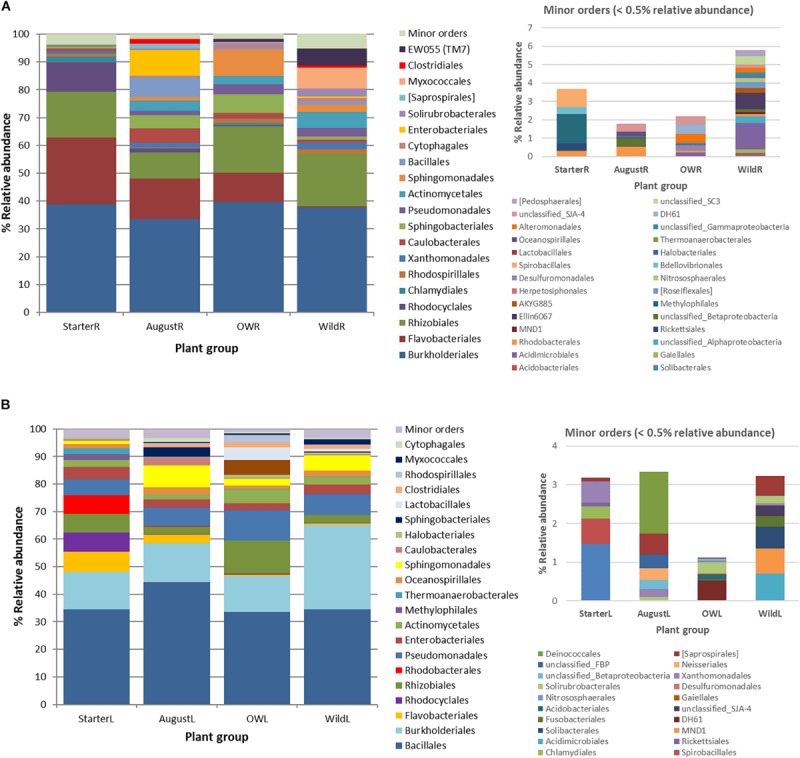
Succession of the major endophytic bacterial taxa (>0.5% average abundance) from **(A)** root samples and **(B)** leaf samples at order level from starter to over-wintered compared with the relative abundance of the community composition in wild plants. Low abundance (minor) orders (<0.5% average abundance) are shown in a separate graph.

The differences in the taxonomic composition of the endophytic bacterial communities between different tissues were also visible on broad taxonomic scales. Leaf communities in all plant groups were dominated by Bacilli (Firmicutes, mainly order Bacillales) along with Alpha-, Beta-, and Gammaproteobacteria (Sphingomonadales, Pseudomonadales), while root communities had high relative abundances of class Bacteroidetes (order Flavobacteriia, mainly in bait plant roots), followed by Alpha- and Betaproteobacteria (Rhizobacteriales and Burkholderiales) (Figures 5A,B). The relative abundances of bacterial orders Bacillales, Pseudomonadales, and Sphingomonadales were significantly higher in the leaves, while Rhizobiales, Burkholderiales, Flavobacteriales, Desulfuromonadales, and Xanthomonadales were relatively more abundant in the roots (Kruskal–Wallis test, *p* < 0.05; Table 1, Supplementary Figures S1A,B).

**TABLE 1:** The bacterial orders with significantly differential enrichment in leaf or root endophyte communities from all plant groups.

**Phylum**	**Order**	**Relative abundance**		**Significance**
		**Leaf**	**Root**	
**Leaf > Root**				
Euryarchaeota	Halobacteriales	0.533	0.025	2.55e−06
Firmicutes	Bacillales	38.02	2.808	1.07e−11
Firmicutes	Lactobacillales	1.343	0.002	1.01e−07
Alphaproteobacteria	Sphingomonadales	5.508	3.342	0.011
Gammaproteobacteria	Oceanospirillales	2.006	0.103	1.36e−11
Gammaproteobacteria	Enterobacteriales	3.215	0.719	8.78e−10
Gammaproteobacteria	Pseudomonadales	7.841	2.166	6.58e−05
**Root > Leaf**				
Bacteroidetes	Flavobacteriales	2.062	11.83	0.001
Bacteroidetes	Sphingobacteriales	2.636	3.561	0.018
Bacteroidetes	[Saprospirales]	0.386	0.661	0.005
Alphaproteobacteria	Caulobacterales	1.349	2.666	0.010
Alphaproteobacteria	Rhizobiales	5.094	15.18	3.28e−07
Betaproteobacteria	Burkholderiales	19.35	37.89	3.49e−07
Gammaproteobacteria	Xanthomonadales	0.128	2.215	<0.001

*The significance level was determined from log transformed relative abundance table using Kruskal–Wallis test (*p* < 0.05). Only bacterial orders with relative abundance >0.5% in the entire dataset were included in the analysis.*

### Endophytic Bacterial Community Structures Are Different in Different Plant Groups and Change in the Field Toward Wild Type Communities

Although plant tissue was the primary determinant of endophyte community structures, as stated above, the plant group (starter, August bait, over-wintered bait, and wild) also significantly impacted the endophytic bacterial community structures (PERMANOVA, plant group: Pseudo-*F* = 5.732, *p* = 0.001; Figure 3A). The community structures were significantly different in different plant groups (PERMANOVA, pair-wise test; Table 2).

**TABLE 2:** Pairwise tests between different plant groups (Permutational MANOVA) testing the effect of plant group on the differences between endophytic bacterial community structures.

**Source**	***dF***	**MS**	**Pseudo-*F***	**Groups**	***t***	***p* (PERMANOVA)**
**Root + Leaf**						
Plant group	3	10326	5.7321	Starter, Wild-August	3.1984	0.001
				Starter, OW	2.5573	0.001
				August, Wild-August	2.4694	0.001
				OW, Wild-August	2.3066	0.001
				Starter, August	2.2888	0.001
				August, OW	1.5866	0.001

**Root**						
Plant group	3	33992	8.2669	Starter, Wild-August	4.1122	0.001
				OW, Wild-August	3.2260	0.001
				Starter, OW	3.0926	0.001
				August, Wild-August	3.0444	0.001
				Starter, August	2.3127	0.001
				August, OW	1.6936	0.001

**Leaf**						
Plant group	3	12312	1.8496	Starter, Wild-August	1.6112	0.001
				Starter, OW	1.4224	0.001
				Starter, August	1.3863	0.001
				OW, Wild-August	1.2784	0.007
				August, Wild-August	1.2469	0.032
				August, OW	1.2366	0.008

*Leaf and root communities of each sample group were either included in the analysis or analyzed separately, as indicated. The *t* value indicates the difference between the groups.*

When communities in different tissues were compared independently, the plant group had a significant impact on community structures of both root and leaf communities, and the community structures shifted to increasingly resemble wild type communities during the experiment (Figures 3B,C). The differences in the structures between root communities were more pronounced (PERMANOVA; Pseudo-*F* = 8.2669, *p* = 0.001) than between the leaf communities (PERMANOVA; Pseudo-*F* = 1.8496, *p* = 0.001). This was also evident in PCoA, where the root communities clustered according to plant group (bait or wild plants) and sampling time (July 2013, August 2013, and July 2014; Figure 3C). This clustering was not clearly visible in the leaf communities (Figure 3B).

### Endophytic Bacterial Community Changes in the Field Result From Species Acquisition and Changes in Relative Abundances, but Not From Species Loss

In order to analyze the changes in community members with time, we compared the OTUs in different plant groups. For this analysis, we combined the bait plant samples from August and over-wintered groups to identify OTUs putatively gained or lost in the field.

Of the total of 196 OTUs analyzed, 34 OTUs were present in only the bait plants, and not detected in wild plant samples (Figure 4B). Fifteen OTUs (3.3% of the total reads) were present in all bait plant groups (starter, August, and over-wintered). Of these, five OTUs represented order Flavobacteriales (Bacteroidetes), which was the most relatively abundant taxon in this group (1.9% of the total reads). 17 OTUs were detected only in the bait plants in the field, with Bacteroidetes (six OTUs) and Alphaproteobacteria (five OTUs) being the most abundant taxa. Two low abundance OTUs representing Deltaproteobacteria and Chloroflexi (0.2% of the total dataset reads), were exclusively present in the starter plants.

Fifty-six OTUs (10.5% of the total reads) were acquired in the field by bait plants and were also present in the wild plants (Figure 4B). Sixteen of these OTUs represented several Actinobacterial orders (mainly Actinomycetales, Solirubrobacterales, and Acidimicrobiales) and 18 OTUs of diverse Proteobacteria with Rhizobiales, and Myxococcales (six and two OTUs, respectively) as the most abundant taxa. Eleven OTUs represented Bacteroidetes, mainly of order Sphingobacteriales, and six OTUs Firmicutes (Clostridiales and Bacillales). Most of the OTUs that were acquired by the bait plants in the field were detected first in the root tissues, as the majority of OTUs (34 out of 73 OTUs) were missing in the August bait plant leaf samples. These OTUs represented bacterial phyla Acidobacteria, Actinobacteria, Bacteroidetes, Firmicutes, Proteobacteria, and TM6. However, five OTUs were only detected in the leaf tissue samples of August bait plants, suggesting acquisition via air (Supplementary Table S1).

A total of 26 OTUs (3.0% of the total reads) were only present in the wild plants. Six of these OTUS were present in both leaves and roots, while 20 OTUs were only detected in roots (Figure 4B). 21 of these OTUs were Proteobacteria: seven OTUs represented Alphaproteobacteria (bacterial families Hyphomicrobiaceae, order Rhizobiales and Rhodospirillaceae, and order Rhodospirillales), five OTUs Deltaproteobacteria (order Myxococcales) and five OTUs Gammaproteobacteria (family Sinobacteraceae, order Xanthomonadales) (Supplementary Table S1).

Finally, most of the OTUs (80 OTUs) were present in all sample types (starter plants, August bait plants, over-wintered bait plants, and wild plants) (Figure 4B). These OTUs constituted 81.4% of the total reads of the dataset and represented 20 bacterial and one archaeal orders in five and one phyla, respectively. However, the relative abundances of these OTUs were highly uneven across plant groups and tissues. As most of the bacterial taxa showed tissue-specific accumulation (see above), we inspected the successional trends in more detail in a tissue-specific manner.

### Successional Trend in the Roots

In total, 191 OTUs found in the root communities represented 10 bacterial and two archaeal phyla, and 48 orders of which 20 orders were present at higher than 0.5% relative abundance (Figure 5A). Analyzed at the order level, 16 orders showed significantly uneven distribution between the plant groups (Kruskal–Wallis test, *p*<0.05; Supplementary Figure S1A). Flavobacteriales (Bacteroidetes) and Rhodocyclales (Betaproteobacteria) were highly abundant (23.9 and 10.5% relative abundance, respectively) in starter plant samples, decreased significantly in the bait plants in the field, and were present at very low relative abundances in the wild plant roots (0.4 and 0.1%, respectively; Figure 5A, Supplementary Figure S1A). In contrast, bacterial orders EW055 (TM7), Clostridiales, Myxococcales, Solirubrobacterales, Cytophagales, Sphingomonadales, and Actinomycetales were significantly less abundant in the starter plants than in the other plant groups and increased in relative abundances in the field bait plants, and were prominent in the wild plant communities (Figure 5A, Supplementary Figure S1A).

### Successional Trend in the Leaves

The total 152 OTUs found in the leaf communities represented two archaeal and seven bacterial phyla. Of the total 21 bacterial orders with higher than 0.5% average relative abundance in the leaf communities, eight orders were unevenly distributed among plant groups (Kruskal–Wallis test, *p* < 0.05; Figure 5B, Supplementary Figure S1B). Flavobacteriales, Rhodocyclales, Rhodobacterales, and Thermoanaerobacterales were significantly more abundant in the starter plants. Sphingobacterales, Rhodospirillales, and Lactobacillales, absent from starter plants, were enriched in the bait plants in the field, but present only at relatively low abundances in the wild plant communities (Figure 5B, Supplementary Figure S1B). On the other hand, Burkholderiales and Pseudomonadales increased in relative abundance in the bait plants, and were abundant in wild plant type communities (Figure 5B, Supplementary Figure S1B).

## Discussion

In this study, plant tissue type was the major determinant of the diversity and community structure of endophytic bacterial communities. This is in agreement with other studies, where the endophytic bacterial communities were shaped by plant tissues and the major endophyte taxa in leaves differed from those in the roots (Bodenhausen et al., 2013; Checcucci et al., 2016; Robinson et al., 2016). However, we detected very few endophyte OTUs restricted to one tissue only, as the majority of OTUs were shared between both tissues, indicating basic ability of most endophytic bacteria, once able to adapt to environment in plant internal tissues, to colonize plant systemically. The divergence of the community structures between tissues in our study resulted thus mainly from differential enrichment of OTUs rather than restriction of OTUs to one tissue only. The leaf endophytic bacterial communities were significantly less diverse than the roots, similar to the findings from winter wheat (*Triticum aestivum*) (Robinson et al., 2016), *Anthurium andreanum* L. (Sarria-Guzmán et al., 2016), as well as from rice (Bertani et al., 2016). Phyllosphere and leaf tissues are known to be relatively nutrient poor environments (Turner et al., 2013). Additionally stressors like desiccation, UV irradiation and the waxy plant cuticle limit the establishment of leaf epiphytes, a major source of leaf endophytes, and restrict colonization of leaf endosphere (Vorholt, 2012). Plants’ above- and belowground compartments also provide chemically different microenvironments, posing an additional selective force for the endophytic bacterial communities (Bai et al., 2015). Checcucci et al. (2016) showed that the leaf endophytic bacterial communities of *Thymus vulgaris* were different from the root and rhizosphere communities, and that the tolerance toward essential oils was higher in the leaf than in the root communities. The essential oils produced in *Thymus* sp., especially by the leaves of *T. vulgaris* are known to possess strong antibacterial activity which could make the environment in the leaves more toxic than in the roots of such plants. Similarly, *O. digyna* leaves contain phenolic compounds, partially secreted by secretory glands, that might limit the colonization of leaf phyllo- and endosphere to highly adapted bacteria.

Phylum Firmicutes and proteobacterial class Gammaproteobacteria dominated leaf communities. The higher prevalence of Firmicutes in the leaves compared to the roots has also been shown in winter wheat (Robinson et al., 2016). The ability of Firmicutes to tolerate radiation and drought (Galperin, 2013) might assist in the colonization in the hostile environment of the leaf surface before accessing the leaf endosphere. Pseudomonadales and Enterobacteriales (Gammaproteobacteria) have been also reported to be prevalent on the leaf surfaces of edible green vegetables including head lettuce, romaine and spinach (Burch et al., 2016), in line with their high relative abundances in leaf endopsheres in this study.

*Oxyria digyna* root communities were highly enriched in OTUs representing Bacteroidetes, especially genus *Flavobacterium*, which were present in high relative abundance, although mainly in the roots of the bait plants. Notably, the major the *Flavobacterium* OTUs dominating bait plant roots were different from those in the wild plant roots (Supplementary Table S1), suggesting that our plants might have acquired these bacteria in the acclimation step, possibly from the tap water used for fertilization and watering the plants when they were acclimatized in the greenhouse and greenhouse outdoor tables. *Flavobacterium* sp. is commonly found in soil and aquatic habitats in warm, temperate, and polar locations (Bernardet and Bowman, 2015). Alternatively, the high relative abundance of *Flavobacterium* sp. in bait root communities may be explained by vertical transmission of endophytic bacteria via seeds and enrichment in micropropagated plants during the micropropagation phase. The majority of *Flavobacterium* sp. can utilize glucose as a carbon and energy source (Bernardet and Bowman, 2015) and since the medium used with the micropropagated plants contained glucose, it was possible that *Flavobacterium* sp. was enriched in the micropropagation phase.

Bacterial orders Burkholderiales (Betaproteobacteria, in particular families Comamonadaceae and Oxalobacteraceae) and Rhizobiales (Alphaproteobacteria) were also highly abundant in the roots, in agreement with Kumar et al. (2017a) who reported that OTUs representing Rhizobiales and Burkholderiales were both part of core microbiome of *O. digyna* root endospheres in three arcto-alpine climate zones. Comamonadaceae are known for their nitrogen fixation ability (Cruz et al., 2001; Kumar et al., 2017b), and were also a major taxon in *nifH*-based diazotroph communities in *O. digyna* (Kumar et al., 2017b) roots, suggesting role in plant nitrogen acquisition in the nutrient poor soils dominating this plant’s main habitats. In particular, *Variovorax paradoxus* (Comamonadaceae) is a common plant symbiont found commonly in rhizosphere and endosphere, and is well-known as a plant growth-promoting bacterial species (PGPR) (Han et al., 2011). OTUs representing Oxalobacteraceae are part of highly conserved core community of *O. digyna* (Kumar et al., 2017a), and isolates from this family have been isolated repeatedly from *O. digyna* root and leaf tissues, seeds and seedlings, indicating tight association with the plant (Nissinen et al., 2012; Given et al., unpublished). The ability of Oxalobacteria to utilize oxalic acid, present in high quantities in *O. digyna* tissues, would make these bacteria well adapted to host plant niche. These isolates have also shown nitrogen fixation ability, as well as ability to solubilize inorganic and organic phosphate (Given et al., unpublished), which could benefit the plants in the nutrient poor soils – in the experimental site soil contains very little soluble nitrate (NO${}_{3}^{-}$), ammonium (NH${}_{4}^{+}$), or phosphate (PO_4_) (Kumar et al., 2016). Intriguingly, Oxalobacteraceae have been also reported as a major endophyte group in *Pinus flexilis* seedlings in nutrient poor soils (Carper et al., 2018).

In addition to plant tissue, plant group significantly shaped the endophytic bacterial community diversity and structure. The biggest differences in endophytic bacterial community diversity and community structure were between starter plants and wild plants in both leaves and roots, and this difference grew smaller the longer the plants were in the field, as the community species richness and diversity increased, and structures shifted toward wild plant communities (Figure 3). Increase in endophytic community diversities resulted from acquisition of new bacteria by bait plans, predominantly via roots from the native soil in the field, in agreement with numerous reports demonstrating that rhizosphere is the main source of endophytic bacteria (reviewed in Hardoim et al., 2015). However, some of the OTUs that were mainly restricted to leaves both in the bait plants and wild plants, were only detected in the leaf tissues, suggesting direct leaf colonization via rain or air. Interestingly, majority of the bacterial OTUs acquired by the bait plants in the field were identical to those detected in the wild plants (Figure 4, Supplementary Table S1), indicating, that the plants are able to assemble a plant species typical endophytic community even when transferred to soil in a developed state. Many of the OTUs acquired in the field represent putative nitrogen fixing taxa, including *Clostridium*, Myxococcales, and Desulfuromonadales (*Geobacter*) (Supplementary Table S1), that have been previously detected in *nifH* targeted study of *O. digyna* (Kumar et al., 2017b), implicating putative role in plant nitrogen acquisition, as well as phosphate solubilization as discussed above for root specific Betaproteobacteria.

We detected virtually no species loss, as only two OTUs with very low relative abundance were present in starter plants only (Supplementary Table S1), and the *Flavobacterium* OTUs dominating bait plants, but not in the wild plants, were not replaced by other *Flavobacterium* OTUs in the field – at least during the duration of this experiment. This suggests, that endophyte communities, once established, are resistant, even in changing conditions. However, our OTU resolution (97%) does not enable differentiation of different bacterial strains or – for some taxa – not even species. Thus any species or strain level turnover would be missed with this approach.

Major part of the endophytic microbiome in this study, however, was shared between all plant groups. This can be explained by colonization of tissue propagated plants (starter plants) in the acclimatization phase in Kuopio or more likely, by vertical transmission of bacteria via seeds and through many cycles of micropropagation. As the micropropagated plants used in this study were obtained from seedlings germinated from surface-sterilized seeds, some of the seed transmitted endophytic bacteria likely survived through the micropropagation phase. This is supported by our observations, that several of the OTUs present wild plants as well as starter plants, including OTUs representing Oxalobacteraceae and Comamonadaceae in this study, were also detected in the core community or seed microbiome of *O. digyna* (Kumar et al., 2017a; Given et al., unpublished). Similar transmission has been repeatedly reported even for plants propagated via meristem cultures (Abreu-Tarazi et al., 2010; Esposito-Polesi et al., 2017). Bacteria in micropropagated plants can have deleterious effects, but they have also been reported to have positive effect on the success of the *in vitro* propagation, for example, in *Prunus avium* (Quambusch et al., 2014; Orlikowska et al., 2017). We detected no bacterial signal from micropropagated plants (in agar, data not shown), indicating very low bacterial yield, but the rich community detected in starter plants suggest bacterial proliferation in plants after transplantation from carbon supplemented and fertilized agar to sand pots and open air. Large overlap between microbial OTUs of greenhouse propagated plants and wild plants of the same species from native soils was also reported by Wagner et al. (2016), with 85% of the OTUS shared between plants grown in greenhouse and potting soil and native field plants. However, the 97% cut-off used for OTU binning likely results in different strains – and in some bacterial genera even different species – being combined in the same OTU complicating the analysis of the origin of these OTUs.

The 26 wild plant specific OTUs, that were not detected in bait plants even after overwintering (Figure 4) represent bacterial families Hyphomicrobiaceae, Rhodospirillaceae, Sinobacteraceae, and unclassified Myxococcales (Supplementary Table S1). These taxa, with the exception of Rhodospirillaceae, have been previously identified as key bacterial taxa in *O. digyna* microbiome and have been shown to be present also in Kilpisjärvi area soils (Kumar et al., 2017a). Thus, we were surprised to see, that these likely important members of *O. digyna* microbiome did not colonize our bait plants. This could be due to duration our experiment (1 year in the field), as the turnover of plant microbiome has been reported to take several years, at least in potting soil grown seedlings (Wagner et al., 2016). Alternatively, these bacteria could be strictly seed-transmitted, or acquired only at the early growth phase of the plant, unable to colonize the developed plant root, already heavily colonized by other bacteria.

Succession has been studied widely in the plant and animal communities as well as soil-associated bacteria. However, little is still known about the succession of plant associated microbial communities. In our study, the bacterial succession in the *O. digyna* endosphere lead to community structures that were strongly shaped by plant tissues and increasingly resembled those of the wild plant communities (Figure 3). This resulted from the acquisition of bacterial endophytes from the environment and changes in the relative abundances of endophytes, but only insignificant species turnover. The change in relative abundances of bacterial taxa was also found to be the major cause for community shift in the developing roots of *Arabidopsis thaliana* by Yuan et al. (2015). The change in *O. digyna* community structures, initiated by plant transfer to field and native soils, likely resulted in strong shift in plant metabolism, involving and impacting both plant–microbe and microbe–microbe interactions via change in endosphere chemical conditions (Schütte et al., 2009; Mengoni et al., 2014). This change favored *O. digyna* core endophytic taxa, with potential for nutrient solubilization and PGPB taxa, likely vital for plants in the nutrient poor native soils and demanding climate. However, there was no elimination of bacterial OTUs (*Flavobacteria* sp.) atypical in wild *O. digyna* plants. Whether this resistance of “alien” endophytes is result from mere priority effect and effective colonization of endosphere by these bacteria, or direct microbe–microbe interaction, requires an experimental and targeted study.

## Data Availability Statement

The datasets generated for this study can be found in the European Nucleotide Archive (ENA) PRJEB21160.

## Author Contributions

RN, CG, and EH conceptualized and planned the study. CG and EH prepared the plant material. CG, RN, and MK performed the field work and analyzed the data. CG did the molecular analyses. RN, CG, and EH prepared the manuscript. All authors participated in the interpretation of the data, edited, and approved the manuscript.

## Conflict of Interest

The authors declare that the research was conducted in the absence of any commercial or financial relationships that could be construed as a potential conflict of interest.

## Supplementary Material

FIGURE S1 The log transformed relative abundances of **(A)** 16 bacterial orders from the root samples and **(B)** eight bacterial orders from the leaf samples with significantly different abundances in the four plant groups (starter bait plants, August bait plants, over-wintered bait plants, and wild plants) (Kruskal–Wallis test, *p* < 0.05). Bacterial orders present in >0.5% relative abundances were included in the analysis.

TABLE S1 Distribution of bacterial OTUs across different tissues **(A)** and different plant groups **(B)** of *Oxyria digyna*.
