# Supplementary figures and images for: Tissue-Specific Dynamics in the Endophytic Bacterial Communities in Arctic Pioneer Plant Oxyria digyna
Source: Front Plant Sci. 2020 May 13;11:561. doi: 10.3389/fpls.2020.00561 (PMC7247849; doi:10.3389/fpls.2020.00561)

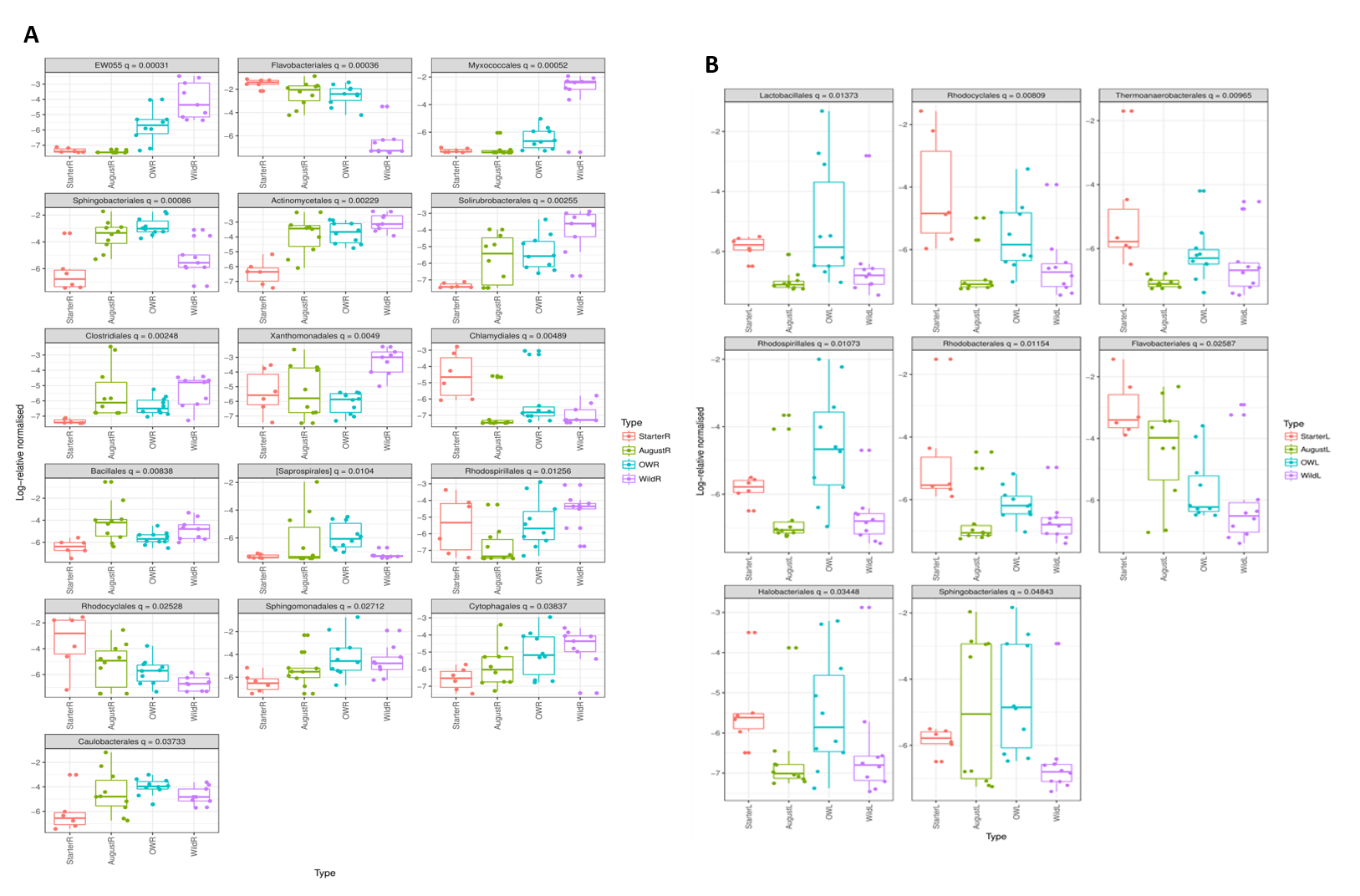

Supplement: FIGURE S1 — The log transformed relative abundances of (A) 16 bacterial orders from the root samples and (B) eight bacterial orders from the leaf samples with significantly different abundances in the four plant groups (starter bait plants, August bait plants, over-wintered bait plants, and wild plants) (Kruskal–Wallis test, p < 0.05). Bacterial orders present in >0.5% relative abundances were included in the analysis. [file Image_1.tif]
